# *Fusarium* Head Blight: Effect of Infection Timing on Spread of *Fusarium graminearum* and Spatial Distribution of Deoxynivalenol within Wheat Spikes

**DOI:** 10.3390/microorganisms9010079

**Published:** 2020-12-30

**Authors:** Elias Alisaac, Anna Rathgeb, Petr Karlovsky, Anne-Katrin Mahlein

**Affiliations:** 1 Institute of Crop Science and Resource Conservation (INRES), Plant Diseases and Plant Protection, University of Bonn, 53115 Bonn, Germany; 2 Molecular Phytopathology and Mycotoxin Research, University of Goettingen, 37077 Goettingen, Germany; anna.rathgeb@agr.uni-goettingen.de (A.R.); pkarlov@gwdg.de (P.K.); 3 Institute of Sugar Beet Research (IfZ), 37079 Goettingen, Germany; mahlein@ifz-goettingen.de

**Keywords:** FHB, *Triticum aestivum*, wheat scab, *Fusarium graminearum*, fungal biomass, deoxynivalenol, deoxynivalenol-3-glucoside

## Abstract

Most studies of *Fusarium* head blight (FHB) focused on wheat infection at anthesis. Less is known about infections at later stages. In this study, the effect of infection timing on the development of FHB and the distribution of fungal biomass and deoxynivalenol (DON) along wheat spikes was investigated. Under greenhouse conditions, two wheat varieties were point-inoculated with *Fusarium graminearum* starting from anthesis until 25 days after anthesis. The fungus and fungal DNA were isolated from the centers and the bases of all the spikes but not from the tips for all inoculation times and both varieties. In each variety, the amount of fungal DNA and the content of DON and deoxynivalenol-3-glucoside (DON-3-G) were higher in the center than in the base for all inoculation times. A positive correlation was found between the content of fungal DNA and DON in the centers as well as the bases of both varieties. This study showed that *F. graminearum* grows downward within infected wheat spikes and that the accumulation of DON is largely confined to the colonized tissue. Moreover, *F. graminearum* was able to infect wheat kernels and cause contamination with mycotoxins even when inoculated 25 days after anthesis.

## 1. Introduction

*Fusarium* head blight (FHB) is a major threat to the yield and quality of wheat worldwide. This is because the kernels of infected plants are light in weight, deformed, and contaminated with a range of mycotoxins [1]. The most susceptible stage of wheat plants for infection with *Fusarium graminearum* is the anthesis stage GS65 [2]. The pathogen can easily penetrate wheat spikes through open florets and extruded anthers. In addition, extruded anthers can trap *Fusarium* spores and stimulate fungal growth by providing nutrients required for germination and penetration [3,4,5]. Various studies have shown that anther extrusion contributed to wheat susceptibility to FHB [6,7].

Francl et al. [8] showed a continuous daily release of *Fusarium* spores from the anthesis stage to the kernels’ soft dough stage in wheat fields adjacent or distant to fields with wheat and maize residues. In fields with residues, the spore deposition on wheat spikes was highly correlated with rain periods. Wheat and maize residues thus represent a significant source of inoculum from anthesis to the later growth stages.

It is generally assumed that wheat spikes are susceptible to *F. graminearum* infection only during anthesis [9]. Under greenhouse conditions, Beccari et al. [10] investigated the effect of the timing of *F. graminearum* infection on disease symptoms, fungal DNA, and secondary metabolites in wheat kernels. They concluded that the timing of infection from 0 to 9 days after anthesis (daa) did not affect disease symptoms, but the infection pressure used in the experiment was too high to allow for this inference (disease severity was 100% in all varieties). Similarly, Siou et al. [2] found the largest amount of fungal DNA and the highest toxin levels after inoculating *F. graminearum* at anthesis or 8 daa. Very low fungal biomass and toxin levels were found in spikes inoculated 18 daa. Under field conditions, Cowger and Arrellano [11] reported comparable disease incidence and deoxynivalenol (DON) levels in wheat kernels resulting from wheat spikes inoculated with *F. graminearum* at anthesis and 10 daa in two experiments. In line with Siou et al. [2], they found very little kernel damage and DON accumulation when *F. graminearum* was inoculated 20 daa. In contrast, Yoshida and Nakajima [12] reported a high incidence of damaged kernels and high toxin levels after infection of susceptible wheat varieties with *F. graminearum* 20 daa. Importantly, late infections may lead to healthy-appearing kernels with high DON levels [11].

Weather conditions, particularly moisture, play an important role in FHB development and DON accumulation in infected kernels. Several researches showed that moisture in late stages after anthesis, i.e., 10, 20, and 30 daa increased disease incidence, disease severity, and DON content [13,14].

Wheat shows several types of resistance to FHB. Resistance to the initial invasion of the spike and resistance to fungal spread from the infection site along the spike are called Type I and Type II resistance, respectively. DON is a major mycotoxin produced by *F. graminearum* and is a virulence factor in FHB [15]. Wheat resistance to DON accumulation by preventing DON synthesis or by detoxification has been originally described as Type III resistance [16] and later reclassified as Type V resistance [15]. Wheat detoxifies DON by glycosylation. DON-3-glucoside (DON-3-G) was reported for the first time in wheat suspension cultures by [17] and later found in naturally and artificially infected wheat kernels [18,19,20]. Lemmens et al. [21] reported the glycosylation of DON by wheat spikes and found that the genetic locus encoding this activity colocalized with a major quantitative trait locus for FHB resistance in wheat. In a comparison of a set of wheat varieties, the authors also showed that FHB resistance and the DON-3-G/DON ratio are closely related. In another study, Lemmens et al. [22] reported that all wheat varieties are able to detoxify DON by glycosylation, which means that this trait has not been recently introduced by breeding for FHB resistance. Moreover, they suggested that increasing FHB resistance in wheat may increase the DON-3-G/DON ratio in kernels.

In the studies of the effect of infection timing on FHB development reviewed above, the entire spikes were spray-inoculated, preventing the investigation of the spread of the pathogen within the spike. The aim of this research was to unravel the effect of the timing of *F. graminearum* infection of the wheat spike on fungal colonization along the spike and on the spatial distribution of DON and DON-3-G.

## 2. Materials and Methods

### 2.1. Plant Material and Experimental Conditions

In this experiment, the two spring wheat varieties (*Triticum aestivum* L.) with different susceptibilities to FHB were: the moderately resistant variety ‘Triso’ (DSV, Lippstadt, Germany) and the susceptible variety ‘Sonett’ (Syngenta, Basel, Switzerland) (Descriptive List of Varieties, the Federal Plant Variety Office, Germany, 2017). A mixture (1:3:6 v/v/v) of sand, horizon c of natural soil [23], and potting substrate ED 73 (Einheitserde, Sinntal-Altengronau, Sinntal, Germany) was used as a growth substrate. The experimental unit consisting of two plants per pot was grown in plastic pots of 12 × 12 × 20 cm in size. The greenhouse conditions were: 16/8 h (day/night) photoperiod obtained from an artificial light with a light intensity of >300 µmol m^−2^ s^−1^ (Philips SGR 140, Hamburg, Germany); 20 ± 2 °C temperature, 50–70% relative humidity (RH), and water on demand [24].

### 2.2. *Fusarium* Inoculum and Inoculation

The isolate S.19 of *Fusarium graminearum* was used from the collection of the Institute of Crop Science and Resource Conservation (INRES), University of Bonn. This isolate was used in previous studies and proved its virulence and ability to produce high amounts of mycotoxins [24]. Fungus culturing and inoculum production were performed according to Alisaac et al. [25]. A Fuchs–Rosenthal chamber was used to adjust the inoculum concentration to 1 × 10^5^ conidia/mL. A fresh inoculum was produced for each inoculation time and used immediately after harvest to inoculate the two central spikelets of the spike. The inoculation was done using a pipette by injecting 5 µL of inoculum and water as a control in the space between the palea and the lemma of the two-terminal florets of the spikelet.

At anthesis, six homogeneous secondary spikes per variety per pot were marked to be inoculated at different dates after anthesis. The inoculation at the anthesis stage GS61 to GS65 was considered 0 daa. Plants of both varieties were inoculated at six different dates (0, 5, 10, 15, 20, and 25 daa; six spikes per variety). Parallel controls of six spikes per experimental unit were mock-inoculated for each inoculation timing. After inoculation, the plants were incubated for 48 h at greenhouse conditions and 95% RH by covering them with plastic bags. At harvest, the spikes were collected, and each spike was divided equally into three parts: tip, center (which contains the inoculated spikelets), and base. Each part was threshed manually.

### 2.3. Pathogen Reisolation

Twelve kernels of each part of the spike (two kernels per part and spike were selected randomly) were superficially sterilized for 2 min in 2% NaOCl and rinsed three times with sterilized distilled water for 2 min each time. The kernels of each part were placed on potato dextrose agar (PDA) (Merck, Darmstadt, Germany) in 9 cm Petri dishes and incubated at 22 ± 2 °C for one week. After incubation, the reisolation ratio was calculated.

### 2.4. DNA Extraction and qPCR

DNA was extracted from 20 mg flour using the cetyltrimethylammonium bromide method with polyethylene glycol precipitation [26] and dissolved in 50 µL TE-buffer (10 mM Tris, 1 mM EDTA, pH 8.0). DNA quality was checked by electrophoresis in 0.8% agarose gel. The DNA samples were diluted 100-fold in water, and *F. graminearum* DNA was quantified by real-time PCR (qPCR) with species-specific primers [27], as described previously [26]. Briefly, the PCR was performed with an initial denaturation at 95 °C for 2 min, followed by 35 cycles of denaturation at 94 °C for 0:30 s, annealing at 61 °C for 0:30 s, and elongation at 68 °C for 0:30 s. Final elongation was carried out at 68 °C for 5 min. To generate melting curves, PCR products were heated to 95 °C for 1 min and cooled to 55 °C for 1 min; the temperature was then slowly raised at 0.5 °C/10 s with continuous fluorescence monitoring. The standards were prepared as 3-fold dilutions of pure *F. graminearum* DNA.

### 2.5. Mycotoxin Extraction and HPLC-MS

For mycotoxin extraction, the flour of each part of the spike was weighed, suspended in acetonitrile/water (84:16) at a ratio of 100 mg flour to 1 mL solvent, and shaken overnight. The mixture was centrifuged at 4500 rpm and 1 mL of the supernatant was transferred to a 2 mL Eppendorf tube and dried under reduced pressure at 40 °C. One milliliter of methanol-1% formic acid in water (25:75, v/v) was used to resuspend the dry residue. The samples were completely dissolved by sonication in an ultrasonic bath. For toxin quantification, an Agilent (Waldbronn, Germany) 1290 Infinity II HPLC system connected to an Agilent 6460 Triple Quad was used. An Agilent Zorbax Eclipse C18 column with 1.8 µm particle size and 100 × 2.1 mm diameter was used for separation [28]. Briefly, the analytes were eluted by a gradient of 5% to 36% methanol in water containing 0.1% formic acid, ionized by electrospray in a positive mode, and detected by tandem mass spectrometry. DON was detected using a precursor ion m/z 297.1 (M+H)^+^ and a product ion m/z 249.1. DON-3-G was detected after in-source fragmentation using the same transition. The limits of detection (LOD) and quantification (LOQ) for DON were 6 and 18 ng/g, respectively. The LOD and LOQ for DON-3-G were 0.11 and 0.36 µg/g, respectively.

### 2.6. Statistical Analysis

Statistical analysis was performed in the open-access software RStudio with the ‘agricola’ package (RStudio, Boston, MA, USA). Kruskal–Wallis test was used to assess the significance of differences in fungal DNA, mycotoxin levels, and DON-3-G/DON ratio at *p* ≤ 0.05, n = 6. Correlations between fungal DNA and mycotoxin levels were assessed using Pearson’s correlation coefficient with the threshold of significance set at *p* ≤ 0.0001, n = 36.

## 3. Results

### 3.1. Pathogen Movement and Reisolation

**Figure 1:**
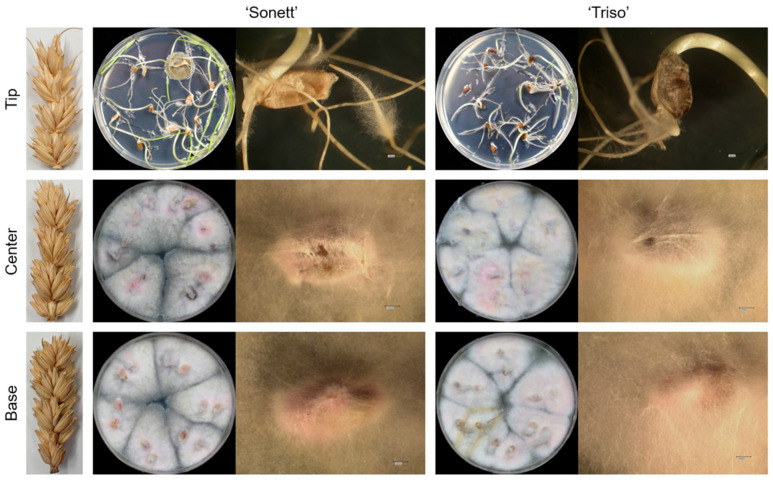
Sampling method and reisolation of *Fusarium graminearum* from the tips (top row), centers (middle row), and bases (bottom row) of the spikes of spring wheat varieties ‘Sonett’ and ‘Triso’ after inoculation of the central spikelets of the spike (n = 12).

The control spikes of both varieties showed no infection in all parts of the spike, i.e., the base, center, and tip. The pathogen grew in the inoculated plants from the inoculation site downward. At all inoculation times and in both varieties, the pathogen was never isolated in the tips of the spikes but 100% from the centers and the bases of the spikes (Figure 1).

### 3.2. Effect of Infection Timing on Fungal DNA Content in Wheat Kernels

**Figure 2:**
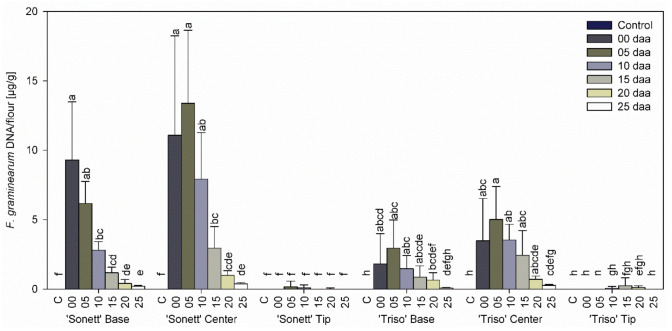
Fungal DNA content in the tips, centers, and bases of the spikes of spring wheat varieties ‘Sonett’ and ‘Triso’ after inoculation of the central spikelets of the spike with *Fusarium graminearum* at different days after anthesis (daa), Kruskal–Wallis test (*p* < 0.0001); n = 6; treatments with the same letters at the level of the variety are not significantly different.

Neither variety contained any DNA of *F. graminearum* in any part of control spikes. In addition, no fungal DNA was detected in the tips of the spikes for all inoculation times in both varieties (Figure 2). The content of *F. graminearum* DNA in the centers of the spikes was higher than in the bases for all inoculation times (Figure 2). In the spike centers of both varieties, the greatest fungal DNA content was observed at the inoculation time 5 daa. The fungal DNA content decreased with inoculation timing until 25 daa. The bases of the spikes of both varieties showed the same trend, except for the maximum DNA level reached in ‘Sonett’ inoculated at anthesis.

### 3.3. Effect of Infection Timing on DON, DON-3-G Content, and Detoxification Ratio in Wheat Kernels

**Figure 3:**
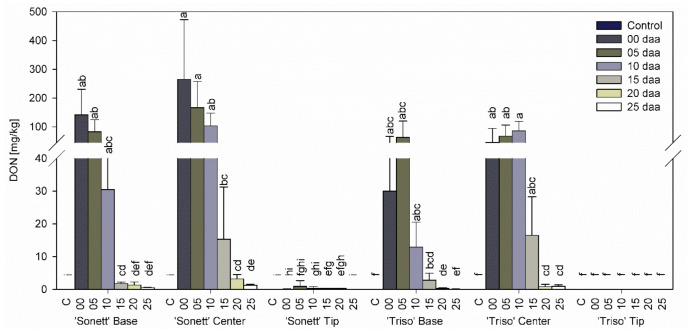
Deoxynivalenol content in the tips, centers, and bases of the spikes of spring wheat varieties ‘Sonett’ and ‘Triso’ after inoculation of the central spikelets of the spike with *Fusarium graminearum* at different days after anthesis (daa), Kruskal–Wallis test (*p* < 0.0001); n = 6; treatments with the same letters at the level of the variety are not significantly different.

**Figure 4:**
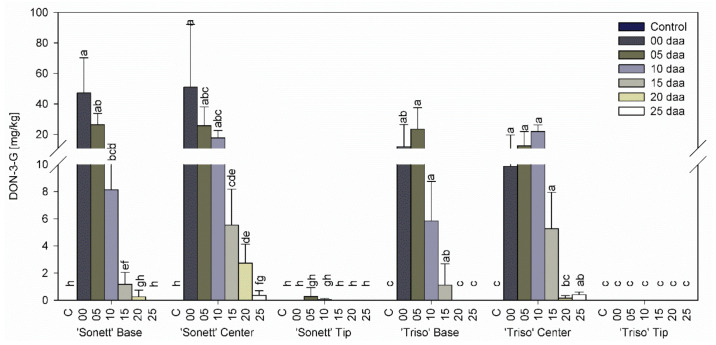
Deoxynivalenol-3-glucoside content in the tips, centers, and bases of the spikes of spring wheat varieties ‘Sonett’ and ‘Triso’ after inoculation of the central spikelets of the spike with *Fusarium graminearum* at different days after anthesis (daa), Kruskal–Wallis test (*p* < 0.0001); n = 6; treatments with the same letters at the level of the variety are not significantly different.

Neither DON nor DON-3-G were detected in any part of control spikes and the tips of inoculated spikes of both varieties at all inoculation times (Figure 3 and Figure 4). In both varieties, higher contents of DON and DON-3-G were shown in the centers compared with the bases at all inoculation times. The content of DON and DON-3-G in the centers and the bases of the spikes of ‘Sonett’ decreased with the inoculation timing from very high levels in spikes inoculated at anthesis to scarcely detectable amounts in spikes inoculated at 25 daa. In the spike bases of ‘Triso’, the highest contents of DON and DON-3-G declined with the inoculation time in a similar way, except that the highest levels accumulated in spikes inoculated 5 daa. In the spike centers of ‘Triso’, the values first rose before they decreased, with the highest levels at 10 daa.

**Figure 5:**
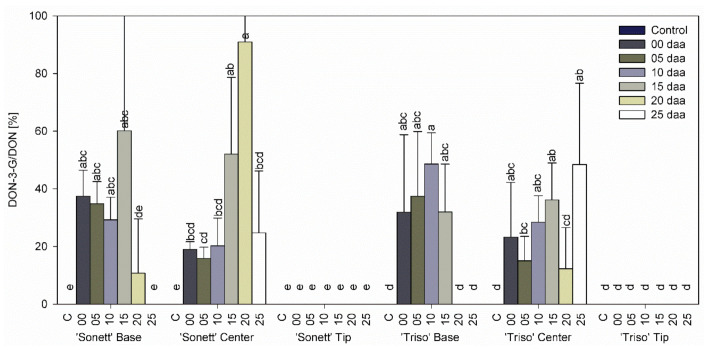
Deoxynivalenol-3-glucoside /deoxynivalenol ratio in the tips, centers, and bases of the spikes of spring wheat varieties ‘Sonett’ and ‘Triso’ after inoculation of the central spikelets of the spike with *Fusarium graminearum* at different days after anthesis (daa), Kruskal–Wallis test (*p* < 0.0001); n = 6. n = 6; treatments with the same letters at the level of the variety are not significantly different.

The ratio DON-3-G to DON, reflecting the detoxification activity, varied between 10 and 90% in the bases and the centers of the spikes in both varieties with a distinct trend (Figure 5). Very low levels or the absence of DON and DON-3-G in the tips of the spikes prevented the assessment of their detoxification activities.

### 3.4. Correlation between Mycotoxin Content and Fungal DNA

**Figure 6:**
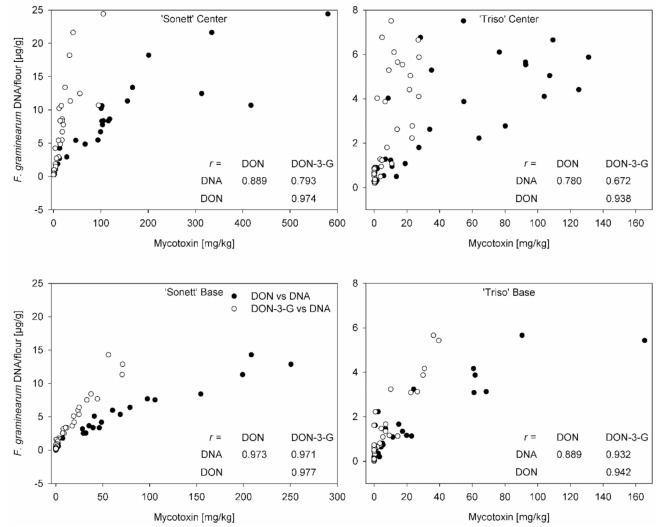
Pearson’s correlation coefficient between fungal DNA and deoxynivalenol, fungal DNA and deoxynivalenol-3-glucoside, deoxynivalenol and deoxynivalenol-3-glucoside in the bases and centers of the spikes of spring wheat varieties ‘Sonett’ and ‘Triso’ after inoculation of the central spikelets of the spike with *Fusarium graminearum* at different days after anthesis (daa) (*p* ≤ 0.0001, n = 36).

Pearson’s correlation coefficient between fungal DNA and mycotoxin level was calculated using the data from all inoculation times. In general, a higher correlation was observed in the bases of the spikes than in the centers for both varieties (Figure 6). The susceptible variety ‘Sonett’ showed a higher correlation than the resistant ‘Triso’ for the same parts of the spike (Figure 6).

## 4. Discussion

The current study showed that the pathogen spreads from the inoculated spikelet downward at all inoculation times. This conclusion is based on the observation that the fungus was not isolated from the tips of the spikes but was present in the centers containing the inoculated spikelets and the bases of both varieties. In addition, fungal DNA was not detectable in the tips of the spikes, while the largest amount of fungal DNA was found in the centers followed by the bases.

The predominantly downward-oriented growth of *F. graminearum* in wheat spikes was reported for the first time in 1905 by the U.S. plant pathologist Edward Freeman [29]. Anatomical and micromorphological details of the infection of spikes were elucidated in microscopic studies for over a century. These studies confirmed that the downward-oriented growth of *F. graminearum* from the point of inoculation was faster than upward-oriented growth [30,31]. The qualitative character of histology, however, prevented quantitative comparisons. Several studies focusing on single spikelets and the neighboring tissues noticed the presence of hyphae below and above the inoculation point without attempting to compare the extent of colonization [32,33]. The most revealing histological study so far regarding the direction of vertical growth of *F. graminearum* in wheat spikes examined ordered sections of the entire point-inoculated spike by optical and electron microscopy [9]. The authors hypothesized that the lack of connection between the vascular vessels of the inoculated spikelet and the rachis above the point of inoculation accounted for the reduced colonization of the upper part of the spike. Another important finding of the study [9] was that two-thirds of the colonized tissue appeared asymptomatic, indicating that visual symptoms do not provide a reliable assessment of infection.

Quantitative studies of the colonization of spikelets above and below the point of inoculation relied on visual symptoms, reisolation of the fungus from individual spikelets, determination of fungal DNA content by qPCR, and indirectly on DON content. Visual symptoms are least reliable because seed infection poorly correlates with visual measurements [34]. Even authors who used visual assessment of spikelet infection in their work reported a conjunction of symptoms caused by progressing infection with the stunting of kernels above the inoculation point due to plugged vascular tissue [35]. The observation that colonized kernels might be asymptomatic [9] further diminishes the utility of visual symptoms for the assessment of infection.

Using reisolation, Argyris et al. [34] established the downward-oriented growth of *F. graminearum* from point-inoculated spikelets for three out of four wheat varieties. Their results were confirmed for two other *F. graminearum* isolates in a greenhouse study [36] and for a large number of *F. graminearum* isolates point-inoculated into the middle of wheat spikes in the field [37]. Visual assessment of point-inoculated spikes of the FHB-resistant variety ‘Alsen’, too, confirmed the downward-oriented spread of *F. graminearum* within the spike. Infected kernels (less than 20 % of all kernels) were found up to three levels below the point of inoculation but only one level above [35].

DON levels were reported for kernels below the point of inoculation in the same study but not above the point of inoculation, apparently due to the low sensitivity of the method (LOD of 20 µg/g) [35]. Similarly, many large amounts of DON were found in spikelets below than above the inoculation point in another study [38]. These results are in line with the findings of the current study (Figure 3). Apparently, transport of DON to upper parts of the spike with the transpiration stream does not significantly affect DON content in kernels (see below).

Can *F. graminearum* colonize wheat spike in an upward direction? Some published results suggest that it might be possible. Argyris et al. [34] reported 100% colonization of spikelets below and above the point of inoculation in their most susceptible accession. When comparing the results from Argyris et al. [34], several aspects have to be considered critically. The breeding line ‘GA 89482-E7’ perhaps possessed lower Type II resistance than the susceptible commercial variety ‘Sonett’ used in the current study. Furthermore, the inoculation conditions differed: Argyris et al. [34] kept inoculated plants at 25–35 °C and 100% humidity for 72 h, while inoculated plants in the current study were kept at 20 °C and 95% humidity only for 48 h. We assume that the combination of high susceptibility of the host plant and highly conducive infection conditions accounted for the heavy infection of spikelets of this particular variety above the point of inoculation. These conditions may occur in some growing areas.

Ha et al. [39] investigated the effect of wheat variety on the hyphal growth of *F. graminearum*. They showed the abundant growth of fungal hyphae in the inoculated spikelet of the susceptible variety ‘Milan’ compared with the resistant ‘Sumai 3′. Fungal DNA amount decreased with infection timing from 5 daa in the centers of both varieties. This tendency was more obvious in the bases of the susceptible variety ‘Sonett’ than in the resistant ‘Triso’. This corresponds to the results of former studies, which showed that the amount of DNA of *F. graminearum* in wheat kernels decreased with infection timing after spray inoculation. Siou et al. [2] reported decreasing amounts of fungal DNA of two aggressive isolates of *F. graminearum* in wheat kernels between 8 daa and 28 daa. Similarly, Beccari et al. [10] described the decreasing accumulation of fungal DNA from 3 daa until 9 daa.

Previous reports documented DON translocation within the plant tissues above the inoculation site. Kang and Buchenauer [40] showed that the hyphae of *F. culmorum* could not be detected in third spikelets above the inoculated spikelet, but DON was present especially in the xylem vessels and phloem sieve tubes, suggesting that DON can be translocated upward through these tissues. DON translocation was also reported after the stem-base-inoculation of bread wheat with different *Fusarium* species, i.e., *F. pseudograminearum*, *F. culmorum*, and *F. graminearum*. Even if the fungus was not present in the spike tissue (including chaff, rachis, and kernels), DON was detectable in the spike tissue of the stem-base-infected plants [41]. In contrast, the results of the current study did not confirm DON translocation from inoculated spikelets into the kernels at the tip of the spike for both varieties and all inoculation times. This is attributed to differences in the investigated tissues since former studies proved an upward DON translocation into spike tissue, including the chaff and rachis, whereas the current study investigated DON translocation in wheat kernels above the inoculation site. In addition, in the stem-base-inoculation, the plants were inoculated at the seedling stage, which means the entire plant tissue was contaminated with DON before spike formation. In the current study, the plants were inoculated at the anthesis stage after spike emergence.

DON content in wheat kernels is affected by several factors, including the wheat variety, infection timing, and weather conditions especially, postanthesis moisture [12,13]. Several studies showed that a significant amount of DON can be produced 20 daa, even when the plants were inoculated at anthesis. In addition, late infection resulted in healthy-appearing kernels contaminated with high levels of DON. The high level of DON was attributed to the long duration of postanthesis moisture that provided optimal conditions for the fungus to induce infection and produce mycotoxins [11,12,14]. This can also be achieved by incubating the plants under plastic bags for 48 h after inoculation. Alisaac et al. [24] showed that incubating wheat plants under plastic bags for 48 h after inoculation resulted in high fungal DNA content and DON contamination even with a concentration of 1 × 10^4^ conidia/mL of inoculum. The current study demonstrated the ability of *F. gra-minearum* to infect and contaminate wheat kernels with DON up to 25 daa. However, the amount of the inoculum that was delivered directly into the space between the palea and the lemma helped to induce the infection even 25 daa. This amount of inoculum will not be available within the wheat spikelets under natural conditions. Therefore, the time window for successful infection under natural conditions is likely to be narrower.

Variety resistance and infection timing had a significant effect on DON content in the kernels. The susceptible variety ‘Sonett’ showed decreasing DON content in the centers and the bases starting from 0 until 25 daa. These results correspond with the results of Siou et al. [2], who reported decreasing DON contents in the highly susceptible wheat variety ‘Royssac’ from 0 to 28 daa. The moderately resistant variety ‘Triso’ exhibited increasing DON contents from 0 to 10 daa in the centers and from 0 to 5 daa in the bases. Beccari et al. [10] reported the same trend of *F. graminearum* to cause higher DON contamination at 3 and 6 daa than 0 and 9 daa in the wheat variety ‘Dyna-Gro Shirley’.

The content of fungal DNA correlated with DON content in both varieties for all inoculation times, which is similar to the results of Siou et al. [2] and Beccari et al. [10]. The correlation appeared tighter in the bases than in the centers of the spikes (Figure 6). We assume that different ages of hyphae in these tissues and a decline of DON production in aging hyphae [33,42] might have accounted for this phenomenon. The centers of the spikes contained young and old hyphae at varying ratios, while the bases were colonized by young hyphae sharing the same DON production rate. The correlation remained rather tight even in the centers of the spikes, indicating that fungal hyphae produced DON at the same rate in all parts of the spike. Hallen-Adams et al. [35] attempted to relate the expression of *Tri*5, which is the key gene of DON synthesis, to DON content in individual spikelets. They found gene expression levels inconsistent with DON levels. For instance, the highest observed DON level was associated with a low *Tri*5 expression, while relatively high *Tri*5 expression was associated with much lower DON levels. The discrepancy is not surprising because DON accumulated in the tissue is the result of DON productivity (corresponding to gene expression) multiplied by the fungal biomass and time rather than to DON productivity alone. Furthermore, the authors used the ratio of threshold cycles (Ct) of *Tri*5 and a housekeeping gene (*GAPDH*) as a measure of relative *Tri5* expression. This is incorrect because Ct is inversely proportional to the logarithm of mRNA level; the difference of Ct values rather than ratio should have been used, as exemplified by the customary ΔΔCt method.

The current study demonstrated that the glycosylation ratio increased as DON content of the wheat kernels decreased at later inoculation times. This was clearly shown in the bases, which contained lower amounts of DON than the centers in both varieties and even stronger in the centers (Figure 5). We speculate that protein synthesis was suppressed by DON in spikelets with higher DON content, reducing the synthesis of the enzyme responsible for the glycosylation. In addition, it was shown that variety resistance plays a role in DON detoxification, with the detoxification ratio being higher for ‘Triso’ compared with ‘Sonett’. This corresponds well with former studies, which showed that DON content and variety resistance are the main factors affecting DON detoxification in wheat [22,43]. The high levels of DON-3-G relative to DON in both varieties show that DON-3-G should be legislated and measured in wheat kernels used in the food and feed supply chain because DON-3-G can be reconverted to DON in humans and animals.

## 5. Conclusions

This study showed that *Fusarium graminearum* is able to infect wheat spikes and cause DON contamination in wheat kernels under greenhouse conditions even when inoculated 25 days after anthesis. The infection timing played a significant role in fungal growth expressed by fungal DNA and DON content in infected kernels. The fungus grew from the inoculation site downward within infected wheat spikes, and DON accumulation was confined to the colonized tissue. Variety resistance and DON content correlated with the glycosylation of DON in infected wheat kernels.
